# Investigation of the Role of Oxidative Stress and Factors Associated with Cardiac Allograft Vasculopathy in Patients after Heart Transplantation

**DOI:** 10.1155/2020/7436982

**Published:** 2020-09-15

**Authors:** Wioletta Szczurek, Mariusz Gąsior, Ewa Romuk, Michał Skrzypek, Michał Zembala, Bożena Szyguła-Jurkiewicz

**Affiliations:** ^1^Silesian Center for Heart Diseases in Zabrze, Poland; ^2^3rd Department of Cardiology, School of Medical Sciences in Zabrze, Medical University of Silesia, Katowice, Poland; ^3^Department of Biochemistry, School of Medical Sciences in Zabrze, Medical University of Silesia, Katowice, Poland; ^4^Department of Biostatistics, School of Public Health in Bytom, Medical University of Silesia, Katowice, Poland; ^5^Department of Cardiac, Vascular and Endovascular Surgery and Transplantology in Zabrze, Silesian Center for Heart Diseases in Zabrze, Poland

## Abstract

Oxidative stress is defined as an imbalance between the production of free radicals and their elimination by the antioxidant defense system. However, the role of oxidative stress in cardiac allograft vasculopathy (CAV) has not been fully understood. Therefore, this study is aimed at determining the role of oxidative-antioxidative balance disturbances in patients after HT. Furthermore, we sought to analyze factors associated with the presence of CAV, with particular emphasis placed on oxidative stress markers. The study analyzed data of 194 consecutive patients after HT who underwent routine visits in the Transplantation Clinic between 2015 and 2016. Total oxidant status (TOS) and total antioxidant capacity (TAC) were measured by the method described by Erel. The oxidative stress index (OSI) was defined as the ratio of the TOS to TAC levels. Patients' mean age was 55.4 ± 15.0 years, and 73.4% were men. The frequency of CAV was 50%. The area under the receiver operating characteristic curves indicated a good discriminatory power of TAC and TOS (0.8940 (0.8515-0.9365); 0.8620 (0.8126-0.9114), respectively) as well as excellent discriminatory power of OSI (0.9530 (0.9279-0.9781)) for CAV detection. Multivariate analysis of the Cox proportional hazard model confirmed that OSI (hazard ratio (HR) = 1.294 (1.204-1.391), *p* < 0.0001), age (HR = 1.023 (1.006-1.041), *p* = 0.0091), and high-sensitivity C-reactive protein (HR = 1.049 (1.016-1.083), *p* = 0.0151) were independently associated with CAV presence. In conclusion, TAC and TOS had a good discriminatory power and OSI had excellent strength for detecting CAV. The independent factors of CAV were higher OSI and CRP levels, as well as older recipient age.

## 1. Introduction

Cardiac allograft vasculopathy (CAV) is a major threat to long-term survival after heart transplantation (HT). The frequency of CAV is estimated to be 8% at 1 year after HT, 30% at the 5-year follow-up, and as high as 50% within 10 years after the procedure [1–3]. Early detection of CAV is very important because cardiac ischemia in HT recipients develops silently without symptoms until cardiac dysfunction or sudden cardiac death occur [1]. The recommended method of CAV detection is routine coronary angiography (CAG); however, it is associated with the use of contrast and ionizing radiation [3]. Intravascular ultrasound-detected intimal thickening is predictive for the development of CAV, but the routine use of this method in the diagnosis of CAV is not recommended. Furthermore, imaging methods can detect CAV in the epicardial arteries, but not in the intramyocardial arteries and veins. Therefore, new noninvasive sensitive and specific tools are necessary for detecting CAV early, which may result in the modification of immunosuppressive therapy, increase in statin doses, and intensive treatment of CAV-related comorbidities. There are numerous immune and nonimmune factors in the donor and recipient that are related to the presence of CAV, but a clear concept of the pathophysiological pathways associated with the development and progression of CAV is still lacking, in particular regarding the role of oxidative stress in CAV development [4, 5]. Oxidative stress is defined as an imbalance between the production of reactive oxygen species (ROS) and the ability of antioxidant mechanisms to neutralize it [4, 6]. Disturbances in the balance of the oxidative-antioxidative system lead to damage to the vascular endothelium, which regulates the flow of macromolecules and cells circulating from the blood to the tissues, increases vascular endothelial permeability, and promotes leukocyte adhesion, which are combined with changes in endothelial signal transduction and transcription-regulated factors, influencing the initiation and progression of CAV [7–9]. The role of oxidative stress in patients with CAV has not been fully understood. Therefore, this study is aimed at determining the role of oxidative-antioxidative balance disturbances in patients after HT. Furthermore, we sought to analyze factors associated with the presence of CAV, with particular emphasis placed on oxidative stress markers.

## 2. Materials and Methods

### 2.1. Study Population and Data Collection

The analyzed group consisted of 194 adult heart transplant recipients without signs of infection or acute rejection who underwent a routine annual visit in the Transplantation Clinic between 2015 and 2016. Exclusion criteria included the following: chronic kidney disease stage 4 or 5, autoimmune and neoplastic diseases, viral hepatitis, chronic pancreatitis or thyroid diseases, cerebral vascular accidents, general surgery, and percutaneous coronary intervention during the last year. Baseline clinical characteristics were collected through a chart review of their medical records. During the visit, echocardiography and a panel of laboratory tests and immunosuppressive drug serum concentrations were performed in all patients. In addition, 10 mL of peripheral blood was collected to determine total oxidant status (TOS) and total antioxidant capacity (TAC) levels. Fasting venous samples were frozen at 80°C for the assay of TAC and TOS levels.

The Medical University of Silesia's local Institutional Review Board approved the study protocol, and all patients provided informed consent. The study was performed in accordance with the ethical standards as laid down in the 1964 Declaration of Helsinki and its later amendments.

### 2.2. Diagnosis of CAV

The diagnosis of CAV was based on the results of CAGs and defined according to the current International Society for Heart and Lung Transplantation (ISHLT) criteria [3]. The group with “early” CAV was defined as the presence of any lesions in the coronary vessels within 2 years of HT, while the “late” CAV group included patients with lesions in coronary vessels confirmed by coronary angiography at least 2 years after HT [3]. The group without CAV was defined as the lack of any lesions in the coronary vessels, while the CAV group included patients from CAV 1 to CAV 3. CAGs were performed electively in all patients starting at 1 year after HT and repeated every 2 years when no lesions were found in the previous study and annually in the presence of lesions. Coronary arteries were visualized in multiple and standardized projections under maximal vasodilation with nitroglycerin (0.1 to 0.2 *μ*g). All patients received intracoronary nitroglycerin before intravenous contrast injection. Presence of CAV was established by consensus of two experienced angiographers.

### 2.3. Treatment

The immunosuppressive regimen consisted of 3 drugs, including mycophenolate mofetil, calcineurin inhibitor (cyclosporine or tacrolimus), and prednisone, which was discontinued at the end of the first year after HT. In the analyzed group of patients, proliferation signal inhibitors and induction therapy were not used. Statins were used in all patients without contraindications or adverse reactions. The use of immunosuppressive medication and statins was recorded at the time of CAV diagnosis or at the time of the most recent angiography for the patients without CAV. At the time of enrollment, all patients were receiving immunosuppressive therapy with calcineurin inhibitor and mycophenolate mofetil and had no acute rejection, clinical signs of infection, or symptoms of acute heart failure.

### 2.4. Laboratory Measurements

The serum TOS level was determined by the method described by Erel [10]. The method is based on the oxidation of Fe2+ ions to form Fe3+ contained in the sample by oxidizing them in an acidic environment and the measurement of the intensity of the complexes of Fe3+ ions with xylenol orange. The absorbance measurement was made at 560 nm (reference 800 nm wave). The calibration method was based on measuring the absorbance of standard solutions of hydrogen peroxide. The determinations were made using a PerkinElmer VICTOR-X3 reader. The TOS concentration expressed in *μ*mol/L. The TAC level was measured by the method described by Erel [11]. This method is based on the decolorization of oxidized ABTS (green color) under the effect of antioxidants contained in the examined material. The concentration was calculated from the standard curve using Trolox as a standard. The absorbance measurement was made at 650 nm using a PerkinElmer VICTOR-X3 reader. The TAC concentration is expressed in mmol/L. In order to evaluate the global balance of oxidation-antioxidant, we calculated the oxidative stress index (OSI), which was defined as the ratio of the TOS level to TAC level.

The complete blood count of patients, as well as hematologic parameters, such as the hemoglobin and hematocrit concentrations, was analyzed using automated blood cell counters (Sysmex XS1000i and XE2100, Sysmex Corporation, Kobe, Japan). Liver and renal function parameters, plasma concentration of cholesterol, and triglyceride and albumin levels were determined with a Cobas Integra 800 analyzer (Roche, Basel, Switzerland). The plasma concentration of fibrinogen was measured using the STA Compact Analyzer (Roche). A highly sensitive latex-based immunoassay was used to detect plasma C-reactive protein (CRP) using the Cobas Integra 70 analyzer (Roche). CRP levels were determined with a typical detection limit of 0.0175 mg/dL.

### 2.5. Statistical Analysis

Statistical analyses were performed using SAS software (version 9.4; SAS Institute, Inc., Cary, NC). Categorical data are presented as numbers with percentages. Continuous data are presented as means (standard deviations) for normally distributed variables or medians with upper and lower quartiles for nonnormal distributions. The chi-square test was used to compare categorical variables. The Shapiro-Wilk test was used to determine whether a random sample came from a normal distribution. The Student *t*-test was used to compare normally distributed continuous variables between the groups. A *p* value < 0.05 was considered statistically significant. Cox proportional hazard regression analysis was used to select the potential factors of CAV detection. Cox's univariable proportional analysis was used to select potential independent predictive factors of CAV for inclusion in the multivariable analysis. The relationship between variables was evaluated using the Spearman rank correlation coefficient. The results are presented as hazard ratios (HRs) with 95% confidence intervals (CIs) and the corresponding values of statistical significance. The diagnostic strengths of the TAC, TOS, and OSI for CAV detection were evaluated by calculating each area under the curve (AUC) from the receiver operating characteristic (ROC) curve analysis. The AUC was computed together with 95% CIs. The ROC curves were quantitatively compared by using the DeLong test, and the optimal cut-off values for the models were determined by using the Youden criterion. The statistical significance between AUC values was tested by the method of Hanley and McNeil. Diagnostic utilities were evaluated using sensitivity, specificity, negative predictive value (NPV), positive predictive value (PPV), negative likelihood ratio (LR-), positive likelihood ratio (LR+), and accuracy.

## 3. Results

Patients' mean age was 55.4 ± 15.0 years, and 73.4% were men. The median time from HT to study inclusion was 8.75 (6.00-12.5) years. The frequency of CAV in the analyzed population was 50%. Median time from HT to CAV detection was 6.00 (3.50-9.50) years. All included patients received optimal immunosuppressive therapy. Details of the clinical characteristics of the analyzed population are provided in Table 1.

To identify risk factors for CAV, patients were categorized into the group with CAV and the group without CAV. There were no significant differences between the analyzed groups in the use of immunosuppressive agents, as well as in terms of the presence of diabetes mellitus type 2, hypertension, lipid metabolism parameters, and antioxidant supplementation. The characteristics of the study population divided into CAV 1 vs. CAV 2/3 groups are presented in Table 2.

**Figure 1:**
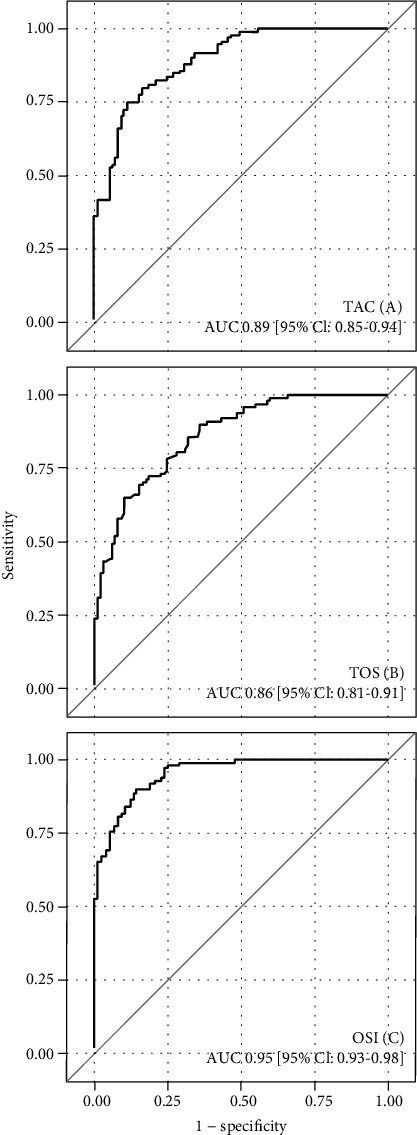
The receiver operating characteristic curves for the TAC (a), TOS (b), and OSI (c).

A ROC curve was generated to determine the accuracy of the TAC level, TOS level, and OSI in CAV detection. The ROC curves for analyzed indicators are presented in Figures 1(a)–1(c).

Results obtained from the ROC analysis for the above parameters are summarized in Table 3.

The AUC indicated a good discriminatory power of the TAC and TOS levels. The cut-off point values for TAC and TOS levels yielded good sensitivity and specificity for association with CAV detection. Both indicators also reached a high PPV and NPV, indicating good results in terms of likelihood ratios as well as good accuracy. The combined assessment of using TAC and TOS levels with the OSI contributed to significantly better detection of CAV (better value of AUC, sensitivity, PPV, NPV, LR+, LR-, and accuracy). The cut-off value of 4.17 for OSI yielded a sensitivity of 89% and specificity of 87%. The difference between the calculated AUC for TAC and OSI levels amounted to 0.0590 (95% CI: 0.1027-0.0153) and that between the TOS level and OSI was 0.0910 (95% CI: 0.1242-0.0578), which were both statistically significant (*p* = 0.0081 and *p* < 0.001, respectively).

The comparison of the area under the ROC curves for the analyzed indicators divided into “early” and “late” CAV are presented in Table 4. There were no significant differences between the AUCs for “early” and “late” CAV in terms of TAC, TOS, and OSI.

**Figure 2:**
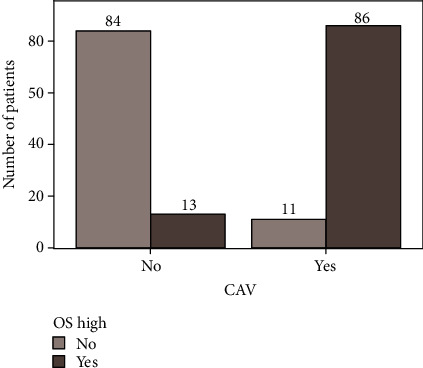
A division into groups with and without CAV in relation to the cut-off value for OSI.

To better illustrate the ability of OSI to separate the patients without CAV from those with CAV, the division into “CAV” vs. “without CAV” in relation to the cut-off value for OSI (4.17) was presented in Figure 2.

Multivariable analysis of the Cox proportional hazard model showed that OSI (hazard ratio (HR) = 1.294 (1.204-1.391), *p* < 0.0001), age (HR = 1.023 (1.006-1.041), *p* = 0.0091), and high-sensitivity C-reactive protein (HR = 1.049 (1.016-1.083), *p* = 0.0151) were independent indicators of CAV. Results of the univariable and multivariable Cox proportional hazard analyses for CAV detection are shown in Table 5.

## 4. Discussion

This study demonstrates that heart transplant recipients with angiographic evidence of CAV can be identified by higher plasma levels of antioxidants and lower levels of oxidants. The holistic assessment of the oxidative-antioxidative balance by the serum OSI allowed us to detect patients with CAV. Moreover, in our study, 2 laboratory parameters, OSI and CRP levels, were independent risk factors for the presence of CAV. In search of improved noninvasive indicators associated with CAV, our study findings could have clinical implications in terms of reducing the number of angiographic procedures. Because high values of OSI and hs-CRP were independent markers of CAV in individual patients, the role of these noninvasive parameters as an ancillary method for exclusion of CAV should be considered.

In accordance with Kargin et al.'s report [4], we observed that in patients with angiographic evidence of CAV, the antioxidant mechanisms were exhausted, as evidenced by a significantly lower TAC level in patients with CAV than in those without CAV. Furthermore, we found increased secretion of ROS, identified by higher values of TOS and OSI in patients with CAV than in those without CAV. TAC and TOS alone had good discriminatory power for CAV detection. The combined assessment of using TAC and TOS with the OSI showed excellent discriminatory power, as well as good sensitivity and specificity for detecting CAV in the analyzed group of patients. These results indicate a shift in the oxidative-antioxidative balance toward prooxidation in patients with CAV.

In physiological conditions, there is a balance between the production and neutralization of ROS by the antioxidant system [12]. Oxidative-antioxidative balance disorders in the prooxidative direction cause a cascade of changes that lead to damage of the vascular endothelium, constituting the main point of CAV development. Permanent smoldering, low-grade vascular inflammation, and oxidative stress cause progressive endothelial damage in the coronary arteries, leading to CAV initiation and progression [4, 5, 7]. To minimize damage caused by ROS, the body uses enzymatic and nonenzymatic antioxidant systems that restore the proper oxidative-antioxidant balance [12]. However after HT, the antioxidant reserve becomes inefficient because of the strong increase in free radical production and their metabolites related to surgery, extracorporeal circulation, episodes of acute rejection, and cytomegalovirus infection [13, 14]. It must be emphasized that the long-lasting decrease of antioxidant defense systems after HT, demonstrated by diminished TOS, can be partly explained by the prooxidant effects of immunosuppressants. Schimke et al. [14], on the basis of analysis of myocardial biopsies, found that HT recipients suffered from oxidative stress in the early- and long-term periods after HT. They concluded that oxidative stress could be associated with CAV, subsequent graft failure, and limited long-term survival after HT [14]. Pechan et al. demonstrated that progressive reduction in the total antioxidant status level in heart transplant recipients occurred very early after HT. It was accompanied by a long-lasting increase of serum ROS levels [13]. The aforementioned study reported strong oxidative-antioxidative balance disorders with a significant long-term decrease of antioxidant reserve in patients after successful HT. Increase in the production of ROS by cells inside or recruited from the graft, along with a decrease in the activity of antioxidant enzymes, may lead to covalent oxidative modification of lipids, proteins, and DNA [7]. In conditions of increased formation of ROS, the heart reacts initially with an adaptational increase in enzymatic antioxidative defense. However, further increase in the formation of free radicals leads to a deficit in the myocardial antioxidative defense. Consequently, increased oxidative stress with an ineffective antioxidant system in the heart leads to a condition characterized by depressed cardiac function [13, 15], causing damage to essential myocardial structures and functions, e.g., the vascular endothelium, myocardial membranes, calcium metabolism, and activity of the beta-receptor-adenylate cyclase system [14]. We found that a sensitive marker of inflammation, the hs-CRP level, was an independent factor of CAV presence. It is postulated that systemic low-grade inflammation plays an important role in CAV development and its progression [16–19]. However, it is unknown whether the inflammation indicated by hs-CRP is the cause or manifestation of the disease. Instead of being an indicator of systemic inflammation, CRP may also have a pathogenetic role. It has been shown that CRP can activate complement and is associated with impaired systemic endothelial vascular reactivity in patients with CAV as well as in patients with native atherosclerosis [17, 20, 21].

Another independent factor associated with the angiographic evidence of CAV was recipient age. It is well known that donor age is an important risk factor for CAV development [5, 22]. However, little is known about the relationship between recipient age and the presence of CAV. The study by Zanchin et al. [23] also showed that the older recipient age was significantly associated with the presence of CAV and significantly higher percent diameter stenosis in coronary vessels. It should be emphasized that recipient age has changed over the years. Currently, the median age at the time of HT has increased by 13 years compared to that in the 90s. Older patients more commonly have a history of cardiac surgery and malignancy, worse renal function, and a greater number of comorbidities [1]. Therefore, it seems that the relationship between recipient age and the presence of CAV is associated with a higher incidence of comorbidities and a greater number of risk factors, which in themselves affect the development of CAV and faster progression of changes in coronary vessels. These results may have clinical implications in that elderly patients after HT should be carefully assessed for the presence of risk factors of CAV, and treatment models should be more aggressive for older patients in order to prevent the development of CAV.

### 4.1. Study Limitation

This study has several limitations. First, in our highly selected group of patients, oxidative stress markers were obtained during different periods after HT. Moreover, we used angiography as a technique of CAV diagnosis, which is the “gold standard” of CAV detection. However, it is known from intravascular ultrasound studies that these results can be misleading in terms of possible underestimation of intimal hyperplasia and vascular remodeling. The use of more sensitive methods of CAV detection, such as intravascular ultrasonography or optical coherence tomography, could strengthen or weaken the relationship between oxidative stress markers and CAV. Additionally, the patients did not routinely undergo baseline (early post-HT) coronary angiography; therefore, donor-derived disease cannot be excluded. However, we reviewed angiographic examinations performed at 1 year after HT, and only 3 patients of the study population had evidence of mild CAV at this time point, suggesting a low prevalence of donor-derived CAV. In addition, we were unable to reliably assess tobacco smoking, which also influences oxidative stress marker levels. To assess the utility of markers of oxidative stress and other factors associated with the presence of CAV in clinical practice, it is necessary to perform prospective external validations in a similar group of patients. Further studies are needed to assess the predictive ability of OSI for recent onset CAV and to predict future CAV. In addition, a relatively small percentage of patients were treated with statins (55%) due to contraindications and adverse reactions. Furthermore, in our study group, blood samples were obtained at different time periods after HT.

## 5. Conclusions

Our study indicates a shift in the oxidative-antioxidative balance toward prooxidation in patients with CAV. TAC and TOS levels had a good discriminatory power for detecting CAV. The holistic assessment of oxidative stress by the OSI with excellent discriminatory power identifies patients with vascular changes. Moreover, our study showed that the OSI, CRP level, and recipient age are independently associated with the presence of CAV. Our results may have clinical importance; incorporating those factors in the management of patients after HT may be potentially helpful in the diagnosis of CAV. Additionally, our study provides new noninvasive, low-cost, and simple indicators for CAV detection. We conclude that the increase in production of ROS accompanied by a reduction in the capacity of the antioxidant defense is associated with the presence of CAV. In addition, this finding may imply that additional therapy with antioxidant substances should be considered an important component of the complex therapeutic program of patients after HT.

## Figures and Tables

**Table 1:** Baseline characteristics of the study population divided into groups without CAV and with CAV.

Parameter	Patients without CAV, *N* = 97	Patients with CAV, *N* = 97	*p*
Donor data			
Age (years)	30.00 (21.00-39.00)	32.00 (22.00-38.00)	0.394
Male (%)	69 (71.1)	74 (76.3)	0.415
Recipient data at the time of study inclusion			
Age (years)	59.00 (37.00-64.00)	62.00 (55.00-67.00)	0.003^∗^
Male (%)	69 (71.1)	74 (76.3)	0.415
Hypertension (%)	71 (73.2)	70 (72.2)	0.872
Type 2 DM (%)	57 (58.8)	63 (64.9)	0.375
Hypercholesterolemia (%)	47 (48.5)	72 (74.2)	<0.001^∗^
BMI (kg/m^2^)	25.00 (22.00-29.00)	27.00 (25.00-30.00)	0.010^∗^
Hemoglobin (mmol/L)	8.80 (8.20-9.40)	8.50 (7.90-9.30)	0.225
Leukocyte count (×10^9^/L)	6.64 (5.51-8.09)	6.38 (5.45-7.67)	0.504
ESR (mm/h)	14.00 (7.00-23.00)	23.00 (12.00-44.00)	0.001^∗^
hs-CRP (mg/dL)	3.40 (1.58-5.40)	5.70 (4.50-7.15)	<0.001^∗^
INR	1.00 (0.97-1.05)	0.99 (0.95-1.05)	0.232
Fibrinogen (mg/dL)	341.00 (298.00-404.00)	396.00 (334.00-469.00)	0.001^∗^
Creatinine (*μ*mol/L)	105.00 (90.00-133.00)	121.00 (99.00-146.00)	0.032^∗^
Bilirubin (*μ*mol/L)	10.00 (7.50-14.60)	10.30 (7.20-14.60)	0.847
Urea (mmol/L)	7.70 (6.10-9.80)	8.90 (7.00-11.60)	0.005^∗^
Uric acid (*μ*mol/L)	406.00 (345.00-453.00)	401.00 (342.00-479.00)	0.818
Total protein (g/L)	74.00 (71.00-78.00)	74.00 (70.00-78.00)	0.992
Albumin (g/L)	46.00 (44.00-48.00)	45.00 (42.00-47.00)	0.005^∗^
HbA1c (%)	5.80 (5.50-6.30)	6.00 (5.50-6.40)	0.505
Sodium (mmol/L)	140.00 (139.00-142.00)	141.00 (139.00-142.00)	0.446
AST (U/L)	22.00 (18.00-26.00)	22.00 (18.00-27.00)	0.688
ALT (U/L)	17.00 (13.00-24.00)	18.00 (15.00-26.00)	0.134
ALP (U/L)	78.00 (62.00-101.00)	91.00 (73.00-110.00)	0.014^∗^
GGTP (U/L)	29.00 (18.00-58.00)	41.00 (25.00-69.00)	0.006^∗^
Cholesterol (mmol/L)	4.55 (3.75-5.29)	4.80 (3.97-5.73)	0.103
Triglyceride (mmol/L)	1.33 (0.92-1.95)	1.43 (1.11-2.12)	0.122
LDL (mmol/L)	2.47 (2.01-3.12)	2.78 (2.12-3.52)	0.164
HDL (mmol/L)	1.45 (0.39)	1.42 (0.44)	0.659
TAC (mmol/L)	1.19 (1.12-1.33)	0.99 (0.91-1.08)	<0.001^∗^
TOS (*μ*mol/L)	3.80 (3.14-4.40)	5.32 (4.48-6.56)	<0.001^∗^
OSI	3.06 (2.51-3.74)	5.44 (4.61-6.76)	<0.001^∗^
LVEF (%)	55.00 (55.00-60.00)	55.00 (52.00-58.00)	0.013^∗^
M. mofetil+tacrolimus (%)	74 (76.3)	70 (72.2)	0.511
M. mofetil %+cyclosporine (%)	23 (23.7)	27 (27.8)	0.511
Statin (%)	44 (45.4)	63 (64.9)	0.006^∗^
Antioxidant supplement (%)	17 (17.5)	13 (13.4)	0.427

Abbreviations: ALP: alkaline phosphatase; ALT: alanine aminotransferase; AST: aspartate aminotransferase; BMI: body mass index; CAV: cardiac allograft vasculopathy; CMV: cytomegalovirus; DM: diabetes mellitus; ESR: erythrocyte sedimentation rate; GFR: glomerular filtration rate; GGTP: gamma-glutamyl transpeptidase; HBA1c: hemoglobin A1c; HDL: high-density lipoprotein; hs-CRP: high-sensitivity C-reactive protein; LDL: low-density lipoprotein; LVEF: left ventricular ejection fraction; M. mofetil: mycophenolate mofetil; OSI: oxidative stress index; TOS: total oxidant status; TAC: total antioxidant capacity. Data are presented as medians (25th–75th percentile), means (standard deviation), or numbers (percentages) of patients; ^∗^*p* < 0.05 (statistically significant).

**Table 2:** Baseline characteristics of the study population divided into CAV 1 vs. CAV 2/3 groups.

Parameter	Patients CAV 1, *N* = 69	Patients CAV 2/3, *N* = 28	*p*
Donor data			
Age (years)	31.00 (22.00-3700)	35.00 (23.50-41.00)	0.735
Male (%)	51 (73.9)	23 (82.1)	0.506
Recipient data at the time of study inclusion			
Age (years)	62.00 (53.00-67.00)	60.00 (55.5-67.50)	0.820
Male (%)	51 (73.9)	23 (82.1)	0.506
Hypertension (%)	48 (69.6)	22 (78.6)	0.657
Type 2 DM (%)	47 (68.1)	16 (57.1)	0.406
Hypercholesterolemia (%)	52 (75.4)	20 (71.4)	0.001^∗^
BMI (kg/m^2^)	27.00 (25.00-30.00)	27.00 (24.5-30.0)	0.940
Hemoglobin (mmol/L)	8.40 (7.80-9.20)	8.95 (7.95-9.45)	0.366
Leukocyte count (×10^9^/L)	6.42 (5.55-7.56)	6.30 (5.19-7.77)	0.811
ESR (mm/h)	22.00 (12.00-42.00)	30.0 (10.50–46.00)	0.574
hs-CRP (mg/dL)	5.65 (4.50-6.90)	6.48 (5.26-7.80)	0.163
INR	0.99 (0.95-1.05)	0.99 (0.95-1.05)	0.883
Fibrinogen (mg/dL)	396.00 (339.00-469.00)	398.5 (321.0-476.50)	0.735
Creatinine (*μ*mol/L)	114.00 (99.00-145.00)	122.50 (95.00-148.00)	0.793
Bilirubin (*μ*mol/L)	10.30 (7.40-14.80)	10.85 (6.75-12.75)	0.491
Urea (mmol/L)	9.20 (7.20-11.40)	8.35 (6.75-12.75)	0.486
Uric acid (*μ*mol/L)	413.00 (356.00–484.00)	389 (318.50-467.00)	0.320
Albumin (g/L)	45.00 (43.00-47.00)	43.00 (42.00-45.00)	0.03^∗^
HbA1c (%)	6.00 (5.50-6.40)	6.10 (5.50-6.75)	0.466
Sodium (mmol/L)	141.00 (139.00-142.00)	141.50 (140.00-142.00)	0.410
AST (U/L)	22.00 (18.00-26.00)	24.00 (18.00-29.00)	0.370
ALT (U/L)	17.00 (15.00-26.00)	22.50 (15.50-28.50)	0.200
ALP (U/L)	88.00 (71.00-109.00)	95.00 (78.00-119.00)	0.153
GGTP (U/L)	42.00 (25.00-75.00)	37.00 (25.50–68.50)	0.741
Cholesterol (mmol/L)	4.71 (3.91-5.75)	5.00 (4.26-5.56)	0.458
Triglyceride (mmol/L)	1.45 (1.17-2.20)	1.42 (1.04-1.96)	0.458
LDL (mmol/L)	2.52 (2.07-3.44)	2.93 (2.20-3.58)	0.496
HDL (mmol/L)	1.34 (1.08-1.68)	1.39 (1.06-1.63)	0.524
TAC (mmol/L)	0.99 (0.90-1.05)	0.98 (0.91-1.10)	0.856
TOS (*μ*mol/L)	5.32 (4.54-6.50)	5.54 (4.48-6.65)	0.889
OSI	5.47 (4.64-6.63)	5.43 (4.41-6.96)	0.921
LVEF (%)	55.00 (51.00-57.00)	55.00 (52.50-58.00)	0.498
M. mofetil+tacrolimus (%)	49 (71)	21 (75.0)	0.742
M. mofetil %+cyclosporine (%)	20 (29.0)	7 (25.00)	0.742
Statin (%)	46 (66.7)	17 (60.7)	0.020
Antioxidant supplement (%)	8 (11.6)	5 (17.9)	0.541

Abbreviations: see Table 1.

**Table 3:** A summary of the receiver operating characteristic curve analysis for TAC, TOS, and OSI.

	AUC (±95 CI)	*p*	Cut-off	Sensitivity (±95 CI)	Specificity (±95 CI)	PPV (±95 CI)	NPV (±95 CI)	LR+ (±95 CI)	LR- (±95 CI)	Accuracy
TAC	0.8940 (0.8515-0.9365)	<0.001	1.08	0.74 (0.64-0.83)	0.85 (0.76-0.91)	0.83 (0.73-0.90)	0.77 (0.67-0.84)	4.8 (2.48-7.12)	0.30 (0.20-0.41)	0.79 (0.73-0.85)
TOS	0.8620 (0.8126-0.9114)	<0.001	4.94	0.65 (0.55-0.74)	0.90 (0.82-0.95)	0.86 (0.76-0.93)	0.72 (0.63-0.80)	6.30 (2.46-10.14)	0.39 (0.28-0.50)	0.77 (0.71-0.83)
OSI	0.9530 (0.9279-0.9781)	<0.001	4.17	0.89 (0.81-0.94)	0.87 (0.78-0.93)	0.87 (0.79-0.93)	0.88 (0.80-0.94)	6,62 (3.21-10.02)	0.13 (0.06-0.20)	0.88 (0.82-0.92)

Abbreviations: AUC: area under the curve; CI: confidence interval; LR-: negative likelihood ratio; LR+: positive likelihood ratio; NPV: negative predictive value; OSI: oxidative stress index; PPV: positive predictive value; TAC: total antioxidant capacity; TOS: total oxidant status.

**Table 4:** A comparison of the area under the ROC curves for the TAC, TOS, and OSI divided into “early” and “late” CAV.

	AUC for “early” CAV (±95 CI)	AUC for “late” CAV (±95 CI)	*p*
TAC	0.8660 (0.7909-0.9411)	0.9033 (0.8603-0.9462)	0.398
TOS	0.8756 (0.8124-0.9389)	0.8575 (0.8029-0.9121)	0.670
OSI	0.9562 (0.9230-0.9894)	0.9520 (0.9245-0.9795)	0.849

Abbreviations: AUC: area under the curve; CAV: cardiac allograft vasculopathy; CI: confidence interval; OSI: oxidative stress index; TAC: total antioxidant capacity; TOS: total oxidant status.

**Table 5:** Univariable and multivariable analyses of CAV indicators.

Parameter	Univariable data		Multivariable data	
	HR (95% CI)	*p*	HR (95% CI)	*p*
Age	1.021 (1.005-1.036)	0.008	1.023 (1.006-1.041)	0.009
BMI	1.058 (1.012-1.106)	0.013		
hs-CRP	1.049 (1.016-1.083)	0.004	1.044 (1.008-1.081)	0.015
ESR	0.1006 (0.999-1.014)	0.107		
OSI	1.298 (1.211-1.392)	<0.001	1.294 (1.204-1.391)	<0.001
Albumin	0.970 (0.919-1.024)	0.270		
ALP	1.003 (0.998-1.008)	0.296		
LDL	1.202 (0.982-1.471)	0.074		
Hypercholesterolemia	1.939 (1.220-3.083)	0.005		

Abbreviations: ALP: alkaline phosphatase; BMI: body mass index; CAV: cardiac allograft vasculopathy; CI: confidence interval; CRP: high-sensitivity C-reactive protein; ESR: erythrocyte sedimentation rate; HR: hazard ratio; LDL: low-density lipoprotein; OSI: oxidative stress index.

## Data Availability

The data that support the findings of this study are available from the corresponding author upon reasonable request.
